# Harnessing m1A modification: a new frontier in cancer immunotherapy

**DOI:** 10.3389/fimmu.2024.1517604

**Published:** 2024-12-02

**Authors:** Xinru Wang, Xiaoqing Ma, Siyu Chen, Minyan Fan, Chenying Jin, Yushi Chen, Shaodong Wang, Zhiying Wang, Fei Meng, Chengwan Zhang, Lin Yang

**Affiliations:** ^1^ State Key Laboratory of Natural Medicines, Jiangsu Key Laboratory of Carcinogenesis and Intervention, School of Basic Medicine and Clinical Pharmacy, China Pharmaceutical University, Nanjing, Jiangsu, China; ^2^ Affiliated Nanjing Jinling Hospital, School of Medicine, Nanjing University, Nanjing, Jiangsu, China; ^3^ Department of Gastroenterology, Qingdao Municipal Hospital, Qingdao, Shandong, China; ^4^ Department of Clinical Laboratory, Jiangsu Province Hospital of Chinese Medicine, Affiliated Hospital of Nanjing University of Chinese Medicine, Nanjing, Jiangsu, China; ^5^ Department of Central Laboratory, The Affiliated Huaian No. 1 People’s Hospital of Nanjing Medical University, Huai’an, Jiangsu, China

**Keywords:** m1A modification, cancer immunotherapy, tumor microenvironment, m1AScore, immune checkpoint inhibitor

## Abstract

N1-methyladenosine (m1A) modification is an epigenetic change that occurs on RNA molecules, regulated by a suite of enzymes including methyltransferases (writers), demethylases (erasers), and m1A-recognizing proteins (readers). This modification significantly impacts the function of RNA and various biological processes by affecting the structure, stability, translation, metabolism, and gene expression of RNA. Thereby, m1A modification is closely associated with the occurrence and progression of cancer. This review aims to explore the role of m1A modification in tumor immunity. m1A affects tumor immune responses by directly regulating immune cells and indirectly modulating tumor microenvironment. Besides, we also discuss the implications of m1A-mediated metabolic reprogramming and its nexus with immune checkpoint inhibitors, unveiling promising avenues for immunotherapeutic intervention. Additionally, the m1AScore, established based on the expression patterns of m1A modification, can be used to predict tumor prognosis and guide personalized therapy. Our review underscores the significance of m1A modification as a burgeoning frontier in cancer biology and immuno-oncology, with the potential to revolutionize cancer treatment strategies.

## 1 Introduction

Epigenetic modifications of RNA refer to chemical modifications that occur on RNA molecules without altering their basic sequence, yet they significantly affect the stability, localization, translation efficiency, and other biological functions of RNA (1, 2). Since the first discovery of RNA modification as a gene expression control mechanism beyond DNA sequence in the 1950s (3), it has become a prominent focus in life science. Researchers have gradually elucidated its regulatory mechanisms and its crucial role in regulating gene expression, cellular differentiation, tissue development, and the onset and progression of diseases. Up to now, more than 170 chemical modifications of RNA have been identified (4).

Common RNA modifications encompass N6-methyladenosine (m6A), N5-methylcytosine (m5C), N1-methyladenosine (m1A), N7-methylguanine (m7G), N4-acetylcytosine (ac4C), pseudouridine (Ψ), uridylation, and adenosine-to-inosine editing (A-to-I), among which m6A is the cutting-edge research domain (5). These modifications are added to RNA by specific “writers” enzymes, removed by “erasers” enzymes, and can be recognized by “readers” proteins, thereby participating in diverse biological processes of RNA (6). In recent years, among the myriad of RNA modifications, the m1A modification has attracted increasing attention. It is a methylation modification of the first nitrogen atom of adenosine. Apart from m6A methylation, m1A methylation is the most prevalent, abundant, and evolutionarily conserved internal post-transcriptional modifications in eukaryotic RNA (7). Furthermore, m1A and m6A have a close relationship—m1A can not only be converted into m6A under alkaline conditions through the Dimroth rearrangement, but also they share some common regulatory factors, like YTHDF1-3 and FTO (8).

**Figure 1:**
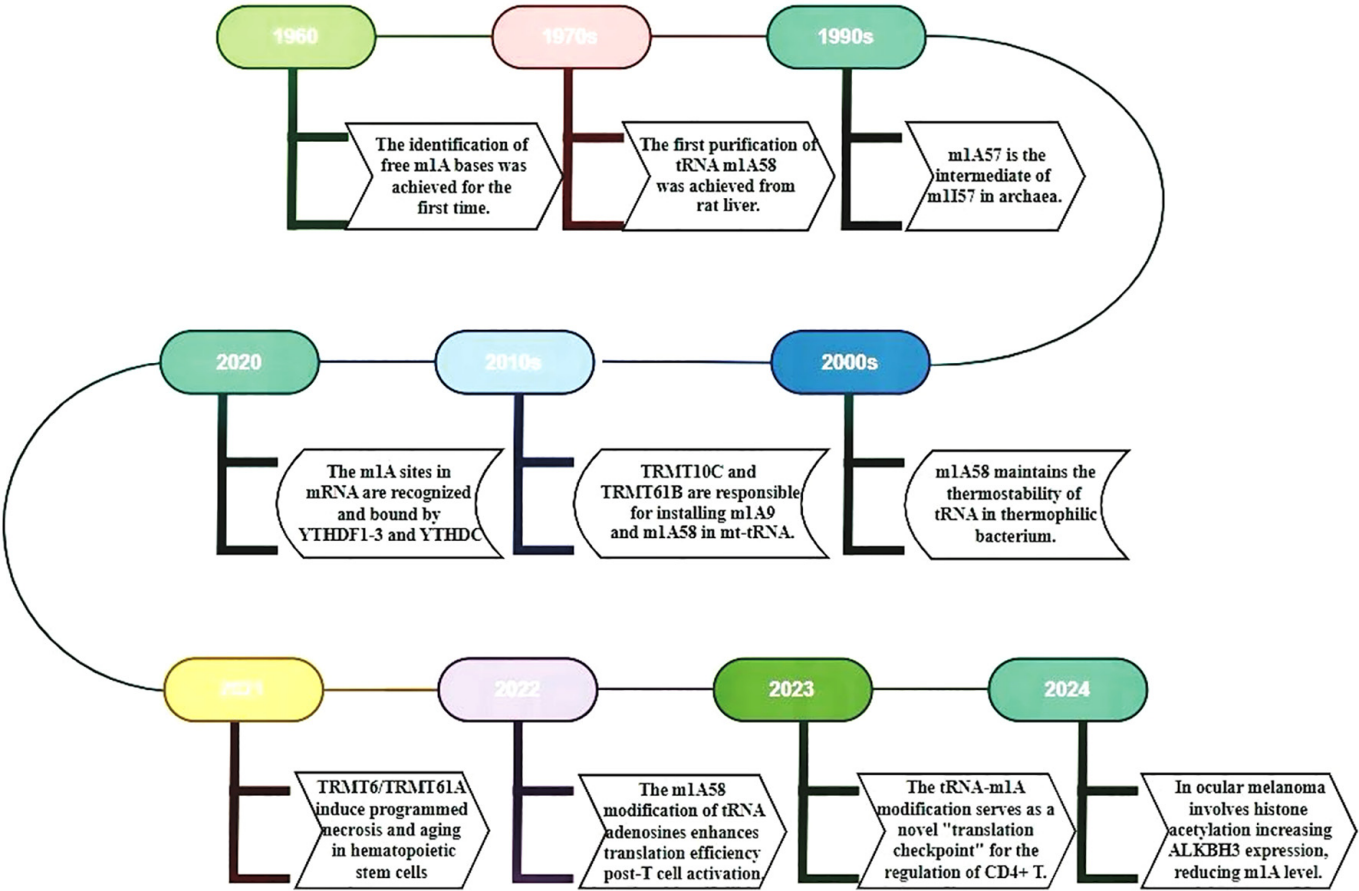
The timeline summarizes key m1A RNA research milestones from 1960 to 2024. It uses different colored boxes to represent key research milestones and discoveries. The timeline provides a detailed account of the evolution of m1A RNA from its initial discovery to a more profound comprehension of its functions. Additionally, it illustrates recent research advancements concerning the functions, regulatory mechanisms, and roles of miRNA in diseases.

First discovered in the 1960s (9), m1A modification has been the subject of research for over half a century (**Figure 1**). With the recent advancements in high-throughput sequencing technology, it has been revealed that m1A modification is ubiquitously present in various types of RNA, such as tRNA, rRNA, lncRNA, and mRNA (10). The detection technologies for m1A modification have been continuously evolving over time, providing critical insights into its biological functions in transcription and translation (11). Especially, single-base resolution detection methods, referring to technologies that precisely identify and quantify specific methylation modifications in RNA molecules at the base level, provides detailed information on gene expression regulation, epigenetics, and disease-related variations (12–14). The Yi research group, leveraging the mismatch caused by m1A during reverse transcription, has developed a high-resolution “m1A-MAP” single-base resolution technology. This technique first enriches RNA containing m1A modifications using m1A antibodies, then employs reverse transcriptase to generate an A-C mismatch when encountering m1A, resulting in a G-A to A-C transition in cDNA. By comparing the mismatch rates of demethylated and untreated samples, m1A modification sites can be precisely located, thus revealing the distribution and abundance of m1A in the transcriptome (15). The Yi group has also developed “m1A-ID-seq,” a novel m1A RNA methylation sequencing technology that combines antibody enrichment with specific enzymatic reactions (7). Both technologies hold significant positions in the detection of m1A modifications. For example, the most commonly used technique is MeRIP-seq/m1A-seq, a methylated RNA immunoprecipitation sequencing method based on antibody enrichment (16). It employs m1A-specific antibodies to enrich RNA fragments with m1A modifications, followed by a high-throughput sequencing to map the precise location and quantify the abundance information of m1A modifications on RNA (17).Recently, Xie et al. has developed m^1^A demethylation editing tool (termed AI-dm^1^A) as well as an m^1^A methylation editing tool (termed AI-m^1^A) by combining the CRISPR/dCas13b system with Chemically Induced Proximity (CIP) technology, enabling the precise and reversible regulation of m1A modification. This tool offers a real-time controllable and reversible means to study m1A dynamics, offering invaluable insights into m1A’s biological functions (18).

Previous reviews on m1A modification have mainly focused on the role of m1A modification in cancer (19), understanding the function of m1A modification in different RNAs and its role in diverse spectrum of malignancies (20). Researches focusing on m1A in the field of cancer immunotherapy are relatively scarce. Cancer immunotherapy has been a significant breakthrough in the context of cancer treatment. It works by activating or enhancing the patient’s own immune system to attack cancer cells and has achieved certain clinical results. However, due to differences in the immune systems and tumor characteristics of different patients, Some patients may not respond or develop tolerance (21). Moreover, the current clinical research evaluation system lacks corresponding methods to assess the durability and special clinical course of immunotherapy (22). Therefore, it is necessary to searching for new targets for cancer immunotherapy continuously (23). The modification of RNA has emerged as a promising direction due to its significant influence on multiple facets of immunotherapy.

This review delves into the significant role and potential of m1A modification in cancer immunotherapy. By revealing how m1A modification affects immune cell function, the tumor microenvironment (TME), and responses to immune checkpoint inhibitor therapy, we provide a scientific basis for developing novel cancer treatment strategies. Furthermore, the concept of m1AScore elaborated in this review may help predict the prognosis of tumor patients and guide clinical treatment decisions, auspiciously improving patients’ treatment outcomes and quality of life. Overall, we emphasize m1A modification as a cutting-edge frontier in the field of cancer biology and immuno-oncology, with the potential to improve approaches to cancer treatment.

## 2 m1a regulators and their biological roles

**Figure 2:**
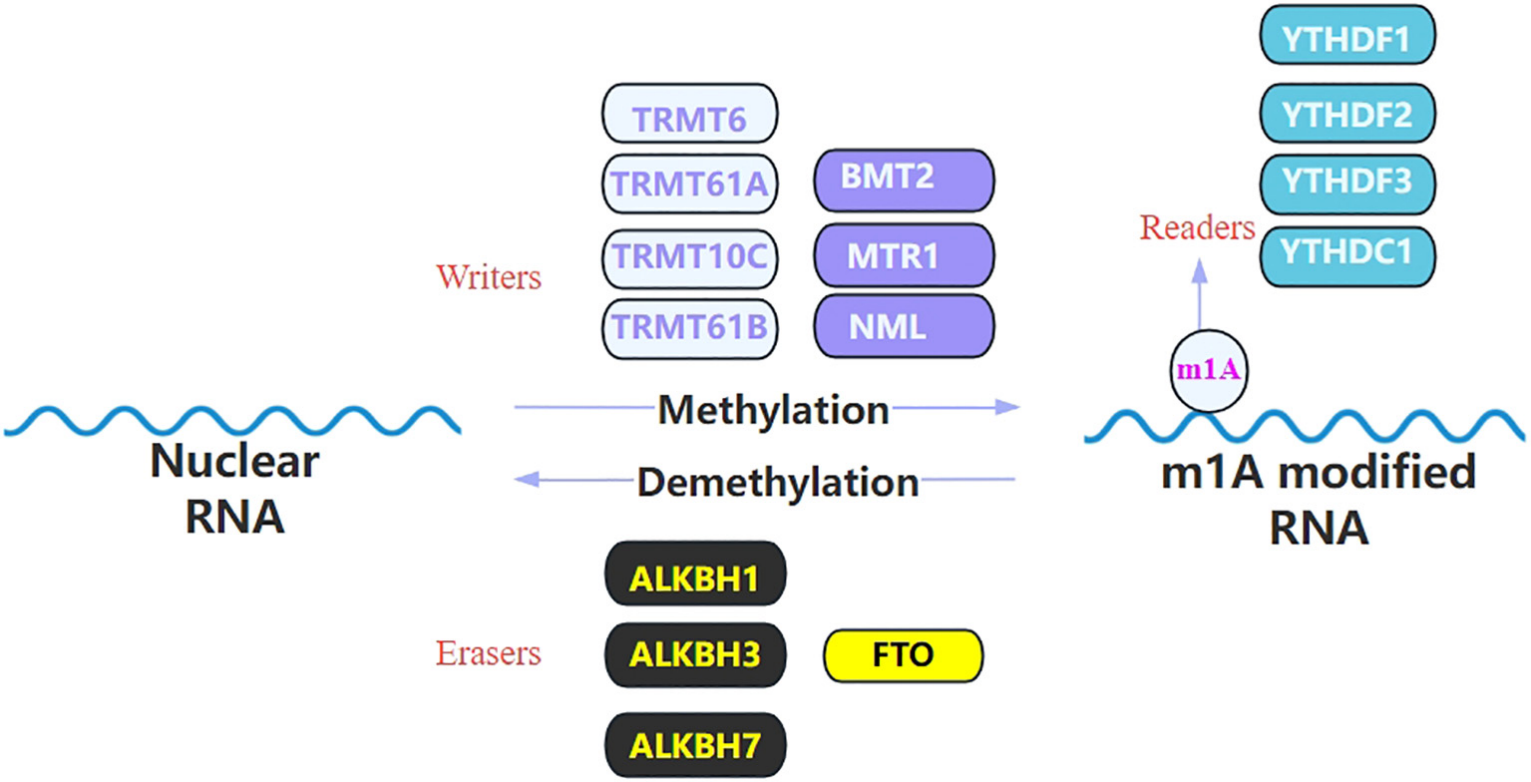
List the identified regulators of m1A modification. m1A RNA modification is catalyzed by the writer and removed by the eraser and it can be recognized by its reader proteins. This image illustrates the m1A modification process of RNA, which is a dynamic regulatory mechanism involving methylation and demethylation. During the methylation process, enzymes known as “writers,” including TRMT6, TRMT61A, TRMT10C, TRMT61B, BMT2, MTR1, and NML, aim to add the m6A modification to RNA molecules. In contrast, “eraser” enzymes such as ALKBH1, ALKBH3, ALKBH7, and FTO are responsible for removing the m1A modification from RNA, thereby achieving demethylation. The modified RNA can be recognized by “reader” proteins, which include YTHDF1, YTHDF2, YTHDF3, and YTHDC1. These proteins participate in the regulation of RNA stability, translation efficiency, and degradation by recognizing the m1A modification.

The modification of m1A is typically enriched in the 5’ UTR region of mRNAs (7), particularly at the first and second positions of the transcripts, as well as near the translation initiation site (17). Additionally, m1A modification is present within the coding sequences, where it is positively correlated with protein synthesis. In some organisms, such as dinoflagellates, m1A modification is predominantly enriched in the 3’ UTR region, where it is negatively correlated with translation efficiency (24). Besides, m1A is also commonly found at conserved sites in tRNA (especially at positions 9, 14, 16, 22, 57, and 58 of tRNA), rRNA and lncRNA (20). Thereby, m1A modification plays an important role in maintaining RNA stability, promoting protein synthesis, and regulating gene expression (1, 2, 25). m1A carries a positive charge under physiological conditions, which may alter the charge distribution of the RNA molecule, thereby affecting its interactions with proteins (10). Additionally, m1A modification disrupts the normal Watson-Crick base pairing, leading to unstable mismatches with other nucleotides. These alterations could potentially impact the secondary structure of RNA and RNA-protein interactions, thereby affecting RNA metabolism processes, including splicing, transport, degradation, and translation (26, 27). The process of m1A methylation involves three types of molecules: “writer”, “eraser” and “reader”, collectively referred to as RNA modification proteins (**Figure 2**). "Writers" are responsible for the methylation of RNA, "erasers" play a role in removing the m1A from RNA, and "readers" can recognize and bind to the m1A-modified transcript and participate in the regulation of downstream biological processes (15, 28).

### 2.1 Writers

Thus far, human cells have been identified six m1A methyltransferases: TRMT6/TRMT61A, TRMT61B, TRMT10C, NML (including RRP8 and RRAM-1 homologues), BMT2, and MTR1 (27, 29). Both TRMT61B and TRMT10C function within the mitochondria (30). TRMT61B is essential for sustaining mitochondrial function and cellular responses to stress, by regulating the methylation of mitochondrial tRNA, thus influencing mitochondrial protein synthesis and overall mitochondrial activity. A reduction in TRMT61B levels can diminish expression of various mitochondrial-encoded proteins, thereby constraining mitochondrial capability, leading to decrease in ATP production, and disruption in oxidative phosphorylation and energy metabolism (31). Additionally, the absence of TRMT61B can lead to senescence in melanoma cell with low levels of aneuploidy, while in melanoma cell with high levels of aneuploidy, it can lead to apoptosis. This may serve as a potential biomarker and therapeutic target for highly aneuploid tumors (32). TRMT10C primarily functions in the methylation of adenosine and guanosine nucleotides at the 9th position of tRNA. Due to the lower GC content of mitochondrial tRNA compared to cytoplasmic tRNA, and the fact that their D-, T-, and variable loops are either absent or of different lengths in supporting the folding of cytoplasmic tRNA, TRMT61C is crucial for ensuring the functional folding and stability of these structurally distinct tRNAs (33).

TRMT61A works together with TRMT6 to form a complex responsible for the m1A modification of mRNA and mitochondrial tRNA, thereby regulating multiple biological processes. Research by He HQ et al. has shown that overexpression of the TRMT6-TRMT61A complex promotes astrocyte senescence through tRNA-m1A58 modification. This modification also induces necroptosis in hematopoietic stem cells (HSCs) by generating 3’-tiRNA-Leu-CAG and activating the RIPK1-RIPK3-MLKL cascade (34), a programmed cell death process mediated by TNF-stimulated signaling (35). Tumor cell-induced necroptosis in endothelial cells facilitates tumor cell extravasation and metastasis (36). Moreover, the specific deletion of TRMT6 in HSCs leads to abnormal expansion and significantly reduced self-renewal capacity in the short term. The tRNA-m1A58 modification also regulates mTORC1 signaling in HSCs to meet their rapid translational demands (37). The overactivation of the mTORC1 pathway in various cancers is widely recognized and is closely associated with cancer cell proliferation, survival, and metabolism (38, 39). Given the critical roles of TRMT6 and tRNA-m1A modification in HSC function, they may serve as potential therapeutic targets for certain hematological malignancies, especially those related to abnormal HSC functions, such as leukemia (40).

### 2.2 Erasers

The erasers of m1A include ALKBH1, ALKBH3, ALKBH7 from the AlkB family, as well as FTO. Among these, ALKBH3 and FTO are the most prominent m1A erasers, making this modification reversible (41). ALKBH3 removes methyl groups from m1A and other alkylated bases (42, 43), modulating key cellular processes like cell cycle regulation and key factors (vascular endothelial growth factor (VEGF), tRNA-derived small RNAs (tDRs) etc.) in the TME. By knocking down ALKBH3, the expression of p21WAF1/Cip1 and p27Kip1, leading to cellcycle arrest at the G1 phase, cellular senescence, and a robust inhibition of cell growth *in vitro* (44). Furthermore, in human urothelial carcinoma cells, ALKBH3 enhances tumor survival, invasiveness, and angiogenesis by modulating the production of reactive oxygen species and the expression of several critical factors like VEGF (45). Additionally, ALKBH3 elevates the sensitivity of tRNA to angiogenin-mediated cleavage, leading to the formation of tDRs. This triggers ribosome assembly and interacts with cytochrome c to prevent apoptosis, thereby promoting cancer progression (46). As for FTO, it can directly inhibit translation by catalyzing m1A tRNA demethylation in both the nucleus and the cytoplasm, thereby suppressing the survival and proliferation of tumor cells (47). This will be further discussed in the following text.

### 2.3 Readers

m1A readers include YTHDF1, YTHDF2, YTHDF3, YTHDC1, all of which belong to YTH family. These proteins can directly interact with m1A-modified RNA molecules through their characteristic YTH domains (48). Compared to the researches on m1A’s “writers” and “erasers”, the study of “readers” has been relatively scarce. Currently, YTHDF3 has been recognized as being able to negatively regulate the invasion and migration of cells. By binding to IGF1R mRNA with m1A modification, YTHDF3 enhances the degradation of IGF1R mRNA, and subsequently reducing the expression of matrix metalloproteinase 9, an enzyme involved in extracellular matrix remodeling and tumor cell invasion (49). With respect toYTHDC1, in addition to its known binding to m6A-RNA, it also binds to m1A-containing RNA after alkylation. YTHDC1, together with the THO complex, prevents DNA breaks induced by nuclear RNA m1A methyltransferases (43). YTHDF2 facilitates the transport of the modified RNA to the P-body via its N-terminal domain, thereby hastening the degradation of the m1A-modified RNA (50). YTHDF1 primarily participates in the metabolism of ATP5D to regulate glycolysis (51).

## 3 Application of m1A RNA modification in tumor immunity

Over the past decade or so, cancer treatment has undergone revolutionary changes. It is no longer limited to traditional therapies that target tumors, such as chemotherapy and radiotherapy (52). With the rapid development and continuous innovation of cancer immunotherapy, more precise and personalized treatments have provided patients with novel therapeutic options and better survival prognoses (53). The main driving force behind this shift is a deeper understanding of the TME. The TME is a complex ecosystem composed of cancer cells, non-cancer cells (including fibroblasts, immune cells, endothelial cells, and vascular cells), as well as extracellular matrix, blood vessels, and nerve fibers, among other non-cellular components (54, 55). The TME not only provides physical support and nutrients for tumor cells but also participates in regulating tumor growth, invasion, metastasis, and response to treatment (56). Additionally, the development of new immunotherapeutic drugs has made a significant contribution to cancer treatment. In particular, the first generation of immune checkpoint inhibitors (ICIs), such as anti-programmed death-1(PD-1)/programmed cell death 1 ligand 1 (PD-L1) and anti-cytotoxic T-lymphocyte-associated protein 4 (CTLA-4) antibodies, can restore the antitumor activity of T cells by blocking immune-inhibitory signaling pathways (57, 58).

As cancer immunotherapy has become a frontier in oncology, understanding how m1A contributes to immune modulation offers new possibilities for treatment strategies. Currently, m1A modification has be recognized as a crucial player in directly affection the behavior of immune cells, and indirectly regulating TME. Additionally, evaluating the expression patterns of multiple m1A regulators in tumor samples can predict tumor prognosis and the state of the TME.

### 3.1 m1A modification and immune cells

#### 3.1.1 m1A modification and T cell

T lymphocytes are the primary effector cells in cellular immunity, producing cytokines to mediate inflammation and regulate other types of immune cells in immune responses (59). Among them, CD4+T cells primarily recognize foreign antigens presented by antigen-presenting cells and mount a response. This response can modulate the activity of other immune cells, such as B cells or CD8+ T cells, and can also initiate new immune responses (60). Upon encountering specific antigens, CD4+T cells rapidly transition from a resting state to an active state, and begin to proliferate and differentiate rapidly (61). This process requires the promptsynthesis of a large amount of functional proteins to meet the demands of bioenergetics and biosynthesis (62, 63). Furthermore, Liu et al. have discovered that the catalytic action of the TRMT6/61 A complex at the 58th site of cytoplasmic tRNA can enhance translation initiation and elongation (64).

**Figure 3:**
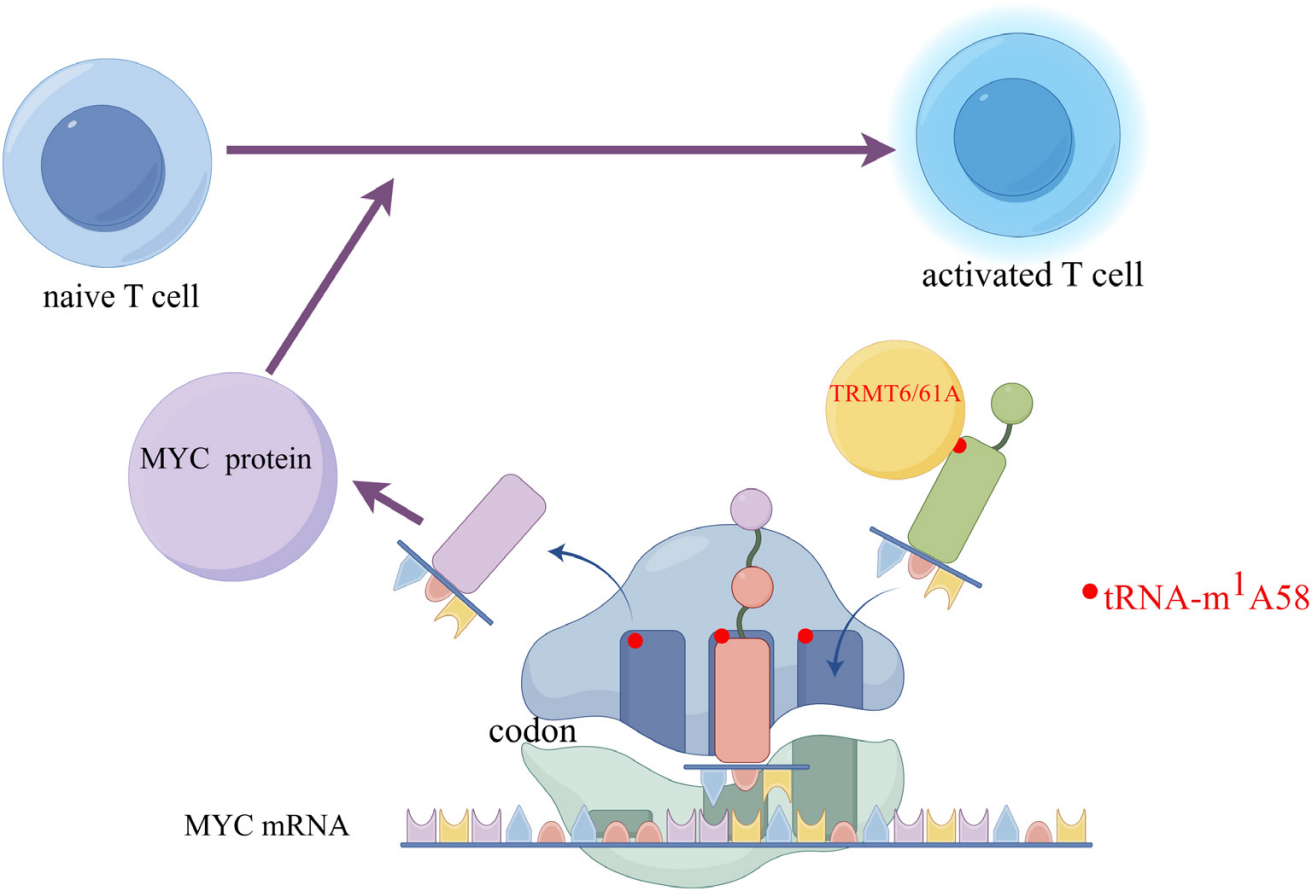
The m1A58 modification in tRNA enhances the efficiency of translation, accelerates the synthesis of the key protein MYC, and promotes the activation of T cells. Created by Figdraw. The left side of the figure shows an initial T cell in an unactivated state. The right side shows an activated T cell, which is the state of T cell activation after receiving specific signals. Myc protein- a transcription factor that plays a key role in cell proliferation, differentiation, and apoptosis is involved in the activation process of T cells. The encoding information of its protein is carried by Myc mRNA. On the mRNA, codons are sequences of three nucleotides that encode specific amino acids. The figure shows ribosomes reading codons on Myc mRNA. Specific tRNA molecules carry the m1A58 modification. This modification is a type of methylation that occurs on tRNA and can affect the stability and translation efficiency of tRNA. TRMT6/61A is a protein complex responsible for adding the m1A58 modification to tRNA. Myc protein affects T cell activation by regulating the translation of mRNA. In initial T cells, Myc protein may regulate translation efficiency by affecting the m1A58 modification of tRNA, thereby influencing the activation process of T cells.

On this established foundation, Li Huabing’s team has uncovered that the m1A modification on tRNA increases translation efficiency, leading to rapid synthesis of key functional proteins such as MYC (65). MYC can regulate the clonal expansion of CD4+T cells by affecting metabolic reprogramming and cell cycle control (66). Consequently, the MYC protein directs naive T cells to transition from a quiescent state to a proliferative one and promotes the swift expansion of activated T cells. Li et al. first observed that during T cell activation, protein translation-related pathways are upregulated, and various tRNAs also exhibit dynamic expression patterns that are upregulated. The tRNA-m1A58 modification enzymes TRMT6 and TRMT61A are also upregulated during the activation process (65). Then they used TRMT6A conditionally knockout mice and found in both *in vivo* and *in vitro* experiments that T cell activation and immune function were impaired, and their proliferative capacity was reduced. It was also discovered that after T cell activation, the translation of various key proteins was hindered, particularly the transcription factor MYC (67). This study suggests that TRMT61A-mediated tRNA-m1A58 modification could serve as a novel “translational checkpoint” for the regulation of CD4+T cell proliferation (**Figure 3**), offering a new RNA epigenetic regulatory strategy for the clinical modification of CD4+T cell functions to treat cancer (67).

#### 3.1.2 m1A modification and macrophages

The impact of m1A modification on immune cells is primarily focused on T cells, with relatively fewer studies on other immune cells. While, still some progress has been made in macrophages. Macrophages can produce a range of cytokines that are crucial for modulating immune reactions, both promoting inflammatory responses and maintaining anti-inflammatory balance. They can also polarize into different phenotypes based on the changing signals from the surrounding microenvironment, adapting to diverse immune demands (68). Besides, macrophages recognize specific molecular patterns of pathogens through their pattern recognition receptors, thereby activating immune responses (69). The following discusses the association between m1A modification and macrophages from two perspectives: cytokines and macrophage polarization.

Research by Woo & Chambers has found that ALKBH3 can enhance the stability of Colony-Stimulating Factor 1 (CSF-1) mRNA. CSF-1 is a cytokine mainly responsible for regulating the generation, survival, differentiation, and function of macrophages (70, 71). Then, CSF-1 activates its receptor CSF-1R to affect the survival, proliferation, migration and invasiveness of cancer cells like breast and ovarian cancer cells. Moreover, increased expression of CSF-1 in breast and ovarian cancer cells has been associated with poor prognosis (72). Therefore, it is possible to explore inhibitors targeting ALKBH3, block the CSF-1/CSF-1R signaling pathway, and develop epigenetic therapies targeting m1A modification to control tumor progression (70). However, further research and clinical trials are needed to translate these findings into clinical applications.

The study of m1A involved in macrophage has also been applied in abdominal aortic aneurysms (AAA). AAA is characterized by the pathological dilation of the abdominal aorta and the continuous weakening of the aortic wall (73). Currently, effective drug treatments are scarce, and surgical repairs pose risks and limitations (74). Infiltration of inflammatory immune cells in the adventitia of the artery is a key characteristic of AAA (75). Strikingly, the transformation of M0 macrophages into pro-inflammatory M1 type or anti-inflammatory M2 type macrophages has a regulatory effect on the vascular inflammation process in AAA (76–78). Moreover, various epigenetic mechanisms are associated with macrophage polarization inspires the exploration and utilization of m1A to modulate macrophage polarization in AAA (79, 80). Research by Wu et al. has provided new insights into the pathogenesis of AAA from the perspective of m1A epigenetic regulation and macrophage polarization (74). The varying expression levels of YTHDF3 acting as “readers” are associated with the infiltration of different immune cells in AAA (80). Using IF double staining analysis, co-expression of YTHDF3 and the macrophage surface marker CD68 was observed in a cell from the adventitia of AAA. Further experiments showed that knockdown of YTHDF3 in M0 macrophages inhibits macrophage M1 polarization but promotes macrophage M2 polarization. Specifically, knockdown of YTHDF3 significantly impaired LPS/IFN-γ-induced macrophage M1 polarization and attenuated the secretion of the inflammatory cytokine IL12, significantly reversing the M0 to M1 polarization of macrophages. Besides, the specific inhibitor of YTHDF3 expression may act as a modulator of macrophage M2 polarization adaptation, which would reduce the secretion of matrix metalloproteinases, promote the repair process of the aortic wall, and alleviate vascular inflammation by downregulating the expression levels of pro-inflammatory cytokines such as IL1β and TNF, and upregulating the secretion of anti-inflammatory cytokines and chemokines such as IL10 and TGFβ, suggesting that YTHDF3 is a potential therapeutic target for AAA (74, 81).

### 3.2 m1AScore in tumor prognosis and immunotherapy

More application of m1A modification in cancer research focuses on analyzing the expression patterns of m1A-related genes to establish m1AScore, which is used to assess prognosis and risk, and guide personalized treatment. Specifically, high-throughput sequencing technologies, such as RNA-seq, are typically employed to collect gene expression data from tumor samples. Genes associated with m1A, including “writers”, “erasers”, and “readers”, are then identified from this data. Subsequent analysis focuses on the expression patterns of these related genes, examining their levels of expression and variations. Statistical methods, such as linear and logistic regression, are utilized to construct a scoring model that predicts the prognosis of cancer patients based on the expression patterns of m1A-related genes. This scoring model is then validated and optimized using independent datasets. Next, by inputting a patient’s gene expression data into the scoring model, an m1AScore is calculated for each individual. Notably, the specific calculation method for the m1AScore may vary across studies, with different research potentially employing distinct sets of genes, statistical approaches, and model-building strategies (82–86). Different scoring systems are employed in various tumor models, which are often also related to immunity, such as the function of immune cells, the response to immunotherapy, and the characteristics of immune cell infiltration in the TME. Therefore, m1A is an indicative biomarker to predict the effectiveness of immunotherapy.

#### 3.2.1 Ovarian cancer (OC)

In the study of ovarian cancer, by comprehensively assessing the m1A modification patterns in 474 OC patients based on 10 m1A regulators and linked them to the immune infiltration characteristics of the TME, Liu et al. found a high m1A score is usually associated with better survival benefits and a lower mutational burden. Moreover, m1A modification affects the TME of ovarian cancer, including the infiltration and composition of immune cells. Researchers identify three distinct m1A modification patterns corresponding to three tumor immune phenotypes: immune desert, immune-inflammatory, and immune-exclusion phenotypes. Tumor patients with an immune-inflammatory phenotype may have a good response to ICIs, while those with an immune-desert phenotype may require other treatment methods to enhance their sensitivity to immunotherapy (85, 87).

#### 3.2.2 Colon cancer

Gao et al. employed m1AScore, which is generated by using profile of expression of the 71 m^1^A-related genes to further demonstrate the m1A patterns in colon cancer They found a low m1AScore is accompanied by enhanced proliferative capacity of CD8+ T cells, increasing the tumor-killing ability of immune cells. Additionally, a low m1AScore is correlated with high microsatellite instability (86), rendering patients have a better response to immune checkpoint inhibitor therapy (88). Moreover, it is also associated with a higher tumor neoantigen burden, which can be recognized by the immune system and elicit an immune response (89). Furthermore, it is related to the expression levels of PD-L1. Therefore, it can be predicted that patients with a low m1AScore will exhibit longer survival times and better treatment responses when undergoing antitumor immunotherapy (86).

#### 3.2.3 Head and neck squamous cell carcinoma (HNSCC)

Wang et al. shed light on the correlation between lncRNAs that harbor modifications of m6A, m5C, and m1A with the survival outcomes, immune contexture, and tumor mutational burden in patients with HNSCC (90). They found m1A modification may affect the stability and function of lncRNAs, which may be involved in the regulation of immune-related gene expression, such as immune checkpoint molecules (91). Moreover, modified RNA influences the composition of immune cells in the TME. The high-risk subgroup may contain a higher number of immunosuppressive cells, such as Regulatory T cells (Tregs) and M2 macrophages, while the low-risk subgroup may have a higher number of immunoactivating cells, such as NK cells and Th1 cells. Thus, by modulating the expression or function of these lncRNAs, it might be possible to enhance the antitumor immune response, thereby improving therapeutic outcomes (90).

#### 3.2.4 Lung cancer

Zhou et al. established a Writer-Score system based on the expression levels of RNA modification writers, such as enzymes related to m1A, m6A, A-to-I, and APA modifications to quantify RNA modification patterns and predict the clinical outcomes of patients with non-small cell lung cancer (NSCLC). These groups of RNA modification patterns show a strong association with various TME characteristics and biological processes. The Writer-Score is also used to predict the prognosis of NSCLC patients receiving neoadjuvant immunotherapy. The study found that patients with a low Writer-Score had a better disease-free survival (p=0,021) and were associated with a better pathological response. Different RNA modification patterns are related to different levels of immune cell infiltration. For example, certain RNA modification patterns are associated with a high level of T helper cells, Tregs, or other immune cells, and the presence of these cells may affect the effectiveness of immunotherapy (92).

#### 3.2.5 Oral squamous cell carcinoma (OSCC)

Three distinct m1A modification patterns were identified in OSCC based on the expression levels of 10 m1A regulators from 502 patients’ samples. These patterns were found to be significantly associated with patient prognosis and the TME characteristics. The cluster with high expression of m1A regulators correlated with lower immune cell infiltration, lower single-sample gene set enrichment analysis (ssGSEA) scores, and higher tumor purity, indicating that m1A modification may influence the formation of TME in OSCC. The expression levels of immune checkpoint molecules such as CTLA-4, PD-1, T cell immunoglobulin and so on, were positively associated with the expression of m1A regulators, immune cell infiltration, and ssGSEA scores (93).

m1Ascore also contributes substantially to pancreatic cancer (94), hepatocellular carcinoma (83), low-grade glioma (84)and other types of cancers. In summary, it shows potential in prognostic research across different cancers and has a certain correlation with immune responses. By combining other clinical parameters, such as tumor mutational burden, m1Ascore can provide more accurate information for personalized treatment and prognostic assessment of cancer patients.

### 3.3 m1A and metabolism regulation

Emerging researches highlight the role of metabolite regulation in enhancing tumor immunotherapy, particularly through modifications like m6A. For example, inhibiting RNA demethylase ALKBH5, has been shown to boost tumor sensitivity to immunotherapy, by downregulating the expression of MCT4/SLC16A3, a lactate transporter, thereby reducing lactate levels in the TME. This metabolic change reduces the presence of immunosuppressive cells like myeloid-derived suppressor cells (MDSCs) and Tregs, ultimately enhancing the tumor’s response to immunotherapy. This discovery highlights ALKBH5 as a potential target for new immunotherapies (95). Although research on the impact of m1A modification on the TME and immune responses is still limited, the findings regarding m6A may provide insights into the effects of m1A modification on metabolic reprogramming and its influence on immune responses (8).

**Figure 4:**
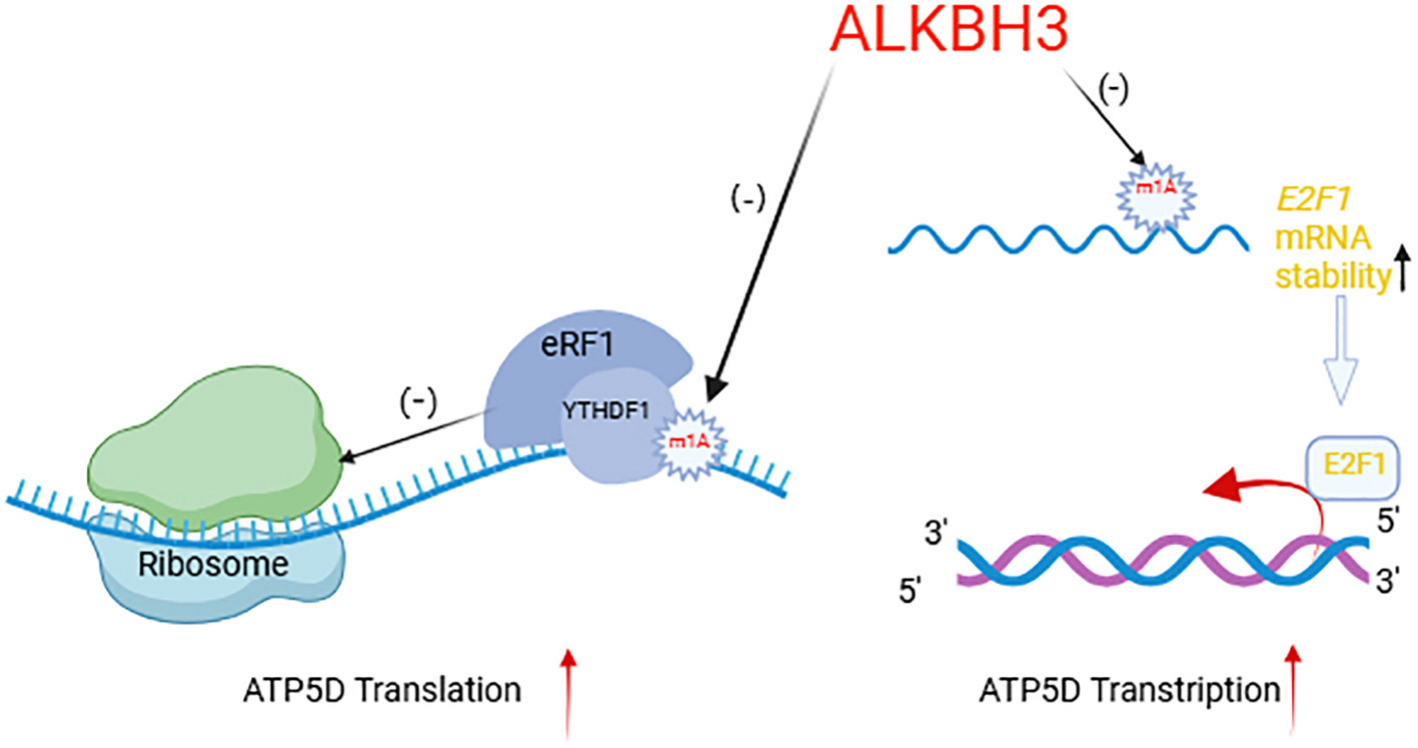
ALKBH3 regulates ATP5D transcription and translation mechanism. Created with BioRender.com. ALKBH3, as a demethylase, specifically targets the m1A modification on mRNA. The arrow pointing to the m1A mark on the mRNA indicates the demethylation process. By removing the m1A, ALKBH3 can affect the stability and translation of mRNA. When the m1A mark is removed, the stability of mRNA increases, as shown by the upward arrow next to “E2F1 mRNA stability,” which may consequently increase the levels of the corresponding protein. The diagram also illustrates the impact of ALKBH3 on translation efficiency. When the m1A mark is bound by the writerYTHDF1 and eRF1 complex, it inhibits translation, as indicated by the negative sign (-). In contrast, after ALKBH3 removes the m1A mark, it allows for more efficient translation, as shown by the positive sign (+) and the upward arrow next to “ATP5D Translation.” In summary, this diagram provides a visual representation of how ALKBH3, through its demethylation activity, can regulate mRNA stability and translation, ultimately influencing protein synthesis.

Wu et al. found that the m1A demethylase ALKBH3 can regulate cancer cell glycolysis through modulating ATP5D, a key subunit of adenosine 5’-triphosphate synthase in two manners (51). On the one hand, the m1A modification at A71 in exon 1 of ATP5D negatively regulates its translation elongation by increasing its binding to the YTHDF1/eRF1 complex, thereby promoting the release of mRNA from the ribosome complex. On the other hand, m1A also regulates the mRNA stability of E2F1, which directly binds to the ATP5D promoter to initiate transcription (96). Overall, ALKBH3 enhances transcriptional and translational efficiency of ATP5D. Targeted demethylation of ATP5D m1A via the dm1ACRISPR system has been shown significantly increase the expression of ATP5D and the glycolysis of cancer cells (**Figure 4**). Other regulatory factors of RNA modification, such as ALKBH5, YTHDF2, and FTO, are also involved in the regulation of glucose metabolism (97).

Wang et al. found that m1A modification mediated by the TRMT6/TRMT61A complex enhances the translation of peroxisome proliferator-activated receptor delta (PPARδ) protein. The activation of PPARδ can promote the expression of genes related to fatty acid oxidation, such as ATP citrate lyase (ACLY) and stearoyl-CoA desaturase 1 (SCD1). It also activates the enzyme 3-hydroxy-3-methylglutaryl coenzyme A reductase (HMGCR) in the cholesterol synthesis pathway to increase cholesterol production. Additionally, PPARδ can affect the uptake and excretion of cholesterol, thereby regulating the levels of cholesterol within the cell (98).

Key enzymes in glycolysis and fatty acid synthesis, such as hexokinase and enolase, as well as fatty acid synthase and acetyl-CoA carboxylase, are targets of m1A modification (97). Tumor cells, by enhancing glycolysis and cholesterol synthesis, may deprive immune cells of the metabolic materials they need, thereby suppressing the function of immune cells. It also alters the metabolic state of the TME, leading to the accumulation of immune-suppressive cells, thus promoting tumor immune evasion. In addition, changes in cholesterol levels affect the expression of immune checkpoint molecules (such as PD-L1) (16, 99, 100).

## 4 m1A modification and ICIs treatments

ICIs are a class of cancer immunotherapies that enhance anti-tumor immune responses by targeting immune checkpoint molecules on the surface of T cells. By blocking the PD-1/PD-L1 and CTLA-4/CD80/86 signaling pathways, they enhance effective immune responses against cancer cells, restore tumor antigen recognition, and ultimately lead to the death of cancer cells (101–103). Although ICIs have achieved significant therapeutic effects in some patients, most patients still experience disease progression after initial treatment. To improve the effectiveness of ICIs, it is crucial to search for new, effective targets and to address issues of resistance (104). A growing number of research highlights the connection between m1A modification and the efficacy of ICIs, such as anti-PD-1 and anti-CTLA-4 therapies.

### 4.1 m1A and PD-L1

Overexpression of MYC protein is closely associated with the occurrence and development of various tumors. However, due to the lack of an enzyme active site pocket suitable for direct action by small molecule drugs, MYC protein is considered an “undruggable” target (105). Recently, Wang et al. reported TRMT61A-mediated tRNA-m1A modification provides a new mechanism and potential therapeutic strategy for the regulation of MYC protein in two ways. First, inhibition of TRMT61A can directly inhibit the proliferation of tumor cells by reducing the synthesis of MYC protein. Furthermore, in tumors treated with oncolytic herpes simplex virus (oHSV), the level of m1A modification increases, leading to reactive upregulation of PD-L1 (106, 107). Therapeutic TRMT61A inhibitors reduce m1A modification, thereby decreasing the *de novo* synthesis of PD-L1, which weakens the immune escape ability of tumor cells and makes them more susceptible to immune system attacks (107). In summary, inhibition of TRMT61A, as a new therapeutic strategy, may improve the sensitivity of tumors to immunotherapy and OV therapy by simultaneously affecting MYC and PD-L1, making it a promising therapeutic target. Future research needs to evaluate the mechanism, efficacy, and safety of TRMT61A inhibitors, in order to provide more effective treatment options for cancer patients.

Moreover, it has been discovered that METTL3, a dual regulator of m1A and m6A, has a close relationship with PD-L1. Ai et al. found that METTL3 can regulate the m6A modification level of PD-L1 in the model of OSCC (108). METTL3 may regulate the transcription or mRNA stability of PD-L1 through m6A modification, thereby affecting the protein level of PD-L1. Knocking down METTL3 reduces the invasion, migration, and proliferation abilities of OSCC cells, and weakens the activation of CD8+ T cells. METTL3 intensifies the metastasis and proliferation of OSCC by regulating the m6A amount of PD-L1, indicating that METTL3 may be a therapeutic target for OSCC patients.

### 4.2 m1A and PD-1

Bao et al. reported that targeting m6A reader YTHDF1 promotes the translation of p65 to upregulate CXCL1, thereby facilitating the migration of MDSCs through the CXCL1-CXCR2 axis (109). The increased MDSCs, in turn, antagonize functional CD8+ T cells in the tumor TME (110). Additionally, depletion of YTHDF1 can reduce tumor growth and enhance anti-colorectal cancer immunity by restoring the infiltration of CD8+ T cells and synergizes with PD-1 blockade to better control tumors (109). Since research has indicated that proteins within the YTH domain family could interact with RNAs that have m1A modifications, possibly serving the role of an m1A reader (48). This opens up research directions for understanding the relationship between m1A modification and the binding of PD-1.

FTO, another regulatory factor shared between m1A and m6A, has been also shown to have a close relationship with PD-1. Yang et al. (111) found that FTO gene expression is upregulated in response to metabolic stress, particularly through the activation of autophagy and the NF-κB signaling pathway. When FTO is knocked down, the methylation level of m6A in key genes that promotes melanoma development, such as PD-1, is increased. This elevated m6A methylation enhances RNA degradation through the action of the m6A reader protein YTHDF2. The reduction of FTO also makes melanoma cells more responsive to interferon gamma (IFNγ) and improves the effectiveness of anti-PD-1 therapy in mice. These findings highlighted the significant role of FTO as an m6A demethylase in the development of melanoma and its resistance to anti-PD-1 treatment. They also suggest that combining FTO inhibitors with anti-PD-1 therapy could potentially overcome resistance to immunotherapy in melanoma patients. Although there is no clear literature showing a connection between m1A modification and PD-1, there are studies have shown that FTO can directly inhibit translation by catalyzing the demethylation of m1A in tRNA (47), therefore, providing a direction for future research.

ICIs therapy has achieved certain successes in cancer treatment. However, primary and acquired resistance limit its clinical application, making it particularly important to explore new treatment strategies to enhance the antitumor effects of immunotherapy (112–114). m1A modification, as a potential mechanism for regulating the expression of immune checkpoints, may become a new target to improve the efficacy of ICI therapy. Currently, the combined application of m1A modification and ICI therapy is still in the research phase. Future research needs to further explore the specific mechanisms of RNA methylation in tumor immunity and develop more RNA methylation regulators, with the hope of achieving breakthroughs in clinical applications.

## 5 Conclusions and prospects

**Figure 5:**
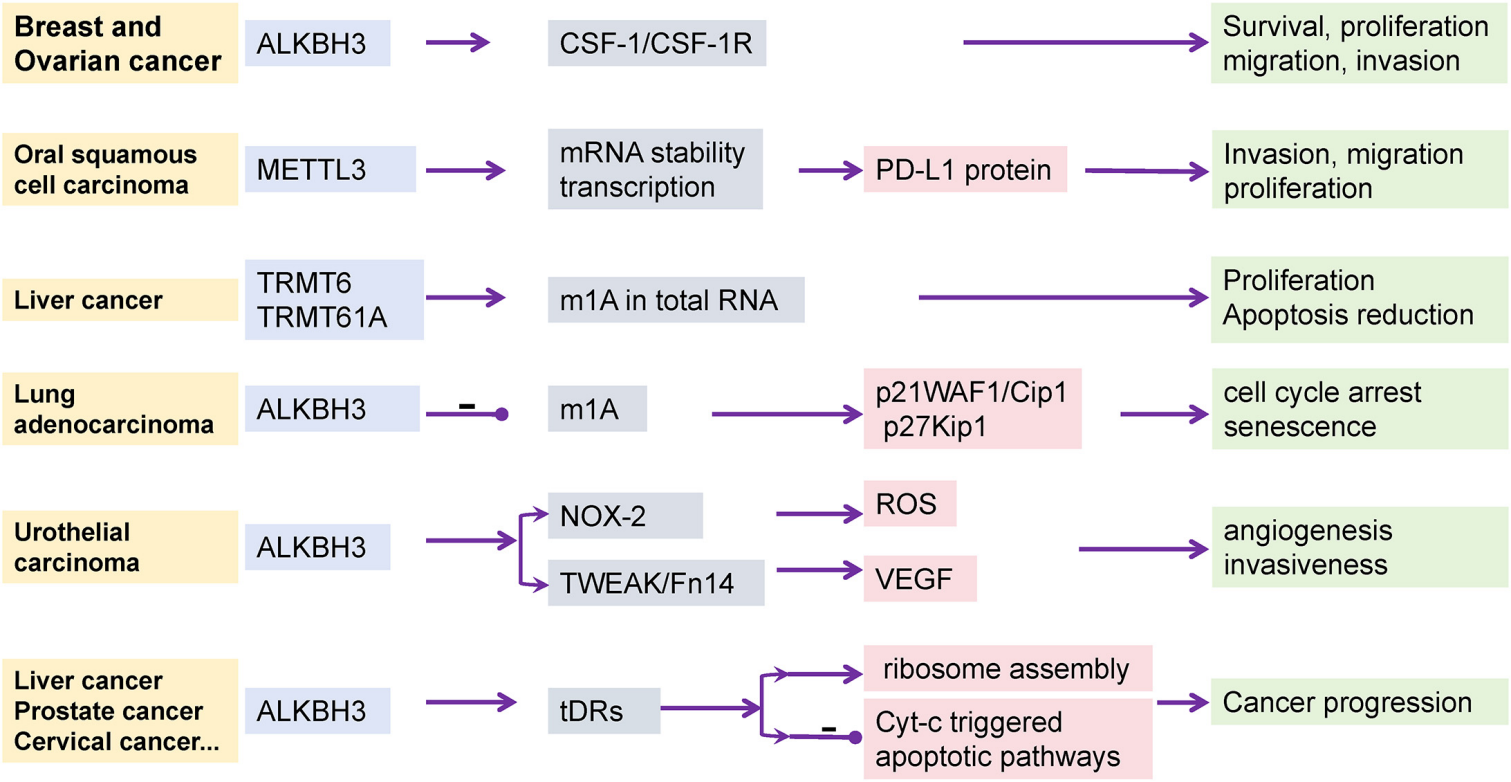
The effect of tumor occurrence and progression by m1A regulators. In different types of cancer, distinct m1A modifications regulate the behavior of tumor cells by affecting specific molecules and signaling pathways. For instance, in breast and ovarian cancers, m1A modifications exert their effects through the CSF-1 signaling pathway; whereas in oral squamous cell carcinoma, m1A modifications influence tumor immune evasion through the expression of PD-L1 protein. The role of ALKBH3 modifications in cancer progression involves multiple levels, including cell cycle regulation, oxidative stress response, and apoptotic pathways, demonstrating the complexity of cancer progression. This figure provides an overview of the role of m1A modifications in different types of cancer and emphasizes its diversity and complexity in tumor biology.

This review initially elucidates the regulatory mechanisms of m1A modification, involving three categories of key enzymes: methyltransferases (writers), demethylases (erasers), and recognition proteins (readers) (15, 28). These enzymes add, remove, or recognize m1A modifications on RNA molecules, participating in the regulation of RNA metabolism and translation processes (24, 25). m1A modification is closely related to the occurrence and development of tumors (**Figure 5**). m1A regulates specific molecules and signaling pathways in various types of cancer, affecting cellular behaviors such as proliferation, migration, invasion, apoptosis, and senescence. Among them, ALKBH3 primarily influences various signaling pathways to regulate the cell cycle and invasiveness of tumor cells (42, 44–46, 70). METTL3 mainly affects RNA stability and regulates the transcription process (108, 115, 116). TRMT6/TRMT61A affects all RNAs, influencing the proliferation and apoptosis processes in tumor cells (31, 32, 34). In the context of tumor immunotherapy, the article emphasizes m1A modification can directly impact immune cell functions (65), such as the proliferation of T cells (60, 65, 67) and the maturation of macrophages (68, 76, 117, 118), and can also indirectly affect immune responses by altering the TME. Furthermore, m1A modification is associated with the responsiveness of tumor cells to immune checkpoint inhibitors (ICIs) (65, 95, 104, 111), such as regulating PD-L1 expression to influence tumor cell immune evasion. This review further introduces the concept of m1AScore, a scoring system based on the expression patterns of m1A modification regulators, used to predict tumor patient prognosis and guide personalized therapy. The m1AScore reflects the overall level of m1A modification in tumor tissues and is closely related to the TME, immune cell infiltration, and patient responsiveness to immunotherapy (83, 85, 86, 94). Additionally, we conclude the role of m1A modification in tumor metabolic reprogramming, indicating that m1A modification may affect immune cell function and tumor microenvironmental metabolic competition by influencing metabolic pathways in tumor cells, such as glucose metabolism and lipid metabolism (51, 98).

Compared with m6A modification, m1A modification still has many areas that have not been fully explored. First, this review has briefly summarized the effects on T cells and macrophages, but there are currently no research results on the role of m1A modification in other immune cells. There is already clear literature explaining the role and mechanism of m6A in immune cells such as NK cells (116), dendritic cells (119), CD8+T cell (115). Therefore, the exploration of its application in immune cells has a certain level of feasibility. In addition, the role of m1A modification in tumor immune escape has not been as specifically reported in dedicated articles as m6A (120).

Although some roles of m1A modification in tumor immunotherapy have been revealed, there are still many potential research directions worth further exploration. With further research and based on the successful cases of m6A, m1A modification may provide new strategies and targets for tumor immunotherapy. Further research is needed to clarify the functions of regulatory factors m1A in gene and protein regulation, especially shared with m6A, and to confirm the clinical utility of m1A modification.

In the research on m6A, it has been reported that there are two main challenges: the scarcity of novel modifications and the promiscuous substrate specificity of many mRNA modifiers. Research is hindered by high error rates, low specificity, and low reproducibility, leading to overestimation or underestimation of modification occurrence (121). There is currently no specific research on drug formulations for m1A modification, but it is likely to face similar challenges. These could all lead to off-target phenomena, such as modifications on tRNA becoming modifications on mRNA. Additionally, the specificity of m1A agents may face other challenges—the selectivity of modification enzymes, as well as subcellular localization. Off-target effects may also lead to some toxic side effects. For example, Zhang et al. explored m1A modifications in mRNA, lncRNA, and circRNA in normal and oxygen-glucose deprivation/reoxygenation-treated mouse neurons, and analyzed the impact of m1A on different RNAs. It was found that m1A may affect the regulatory mechanisms of non-coding RNAs, such as the interaction between lncRNA and RNA-binding proteins, and the translation of circRNA. m1A modification also mediates the competing endogenous RNA (ceRNA) mechanism of circRNA/lncRNA-miRNA-mRNA, and modification in the 3’UTR of mRNA can hinder the binding of miRNA to mRNA. As a result, m1A modification affects the formation and function of synapses, thereby affecting neural transmission and communication between neurons, and subsequently altering neuronal survival, apoptosis, and autophagy (122, 123). Fortunately, the application of computer-aided design and gene editing technologies may help improve this issue. For example, studies have shown that using genome editing technologies such as CRISPR/Cas9 (124) or CRISPR-Cas12a (125) can precisely knock out or knock in specific m1A modification sites to study their function and the specificity of drugs.

With a deeper understanding of the role of m1A modification in cancer immunotherapy, it is anticipated to become a new target for cancer treatment, providing a scientific basis for the development of new immunotherapeutic strategies. Future research will continue to explore the mechanisms and clinical applications of m1A modification, aiming to achieve more precise and effective cancer treatments.
